# *In Silico* Analysis of the *Quorum Sensing* Metagenome in Environmental Biofilm Samples

**DOI:** 10.3389/fmicb.2018.01243

**Published:** 2018-06-07

**Authors:** Jorge Barriuso, María J. Martínez

**Affiliations:** Centro de Investigaciones Biológicas, Consejo Superior de Investigaciones Científicas, Madrid, Spain

**Keywords:** *quorum sensing*, Lux-genes, AI-2, metagenomes, biofilms

## Abstract

*Quorum sensing* (QS) is a sophisticated cell to cell signaling mechanism mediated by small diffusible molecules called “autoinducers.” This phenomenon is well studied in bacteria, where different QS systems are described that differ between Gram-negative and Gram-positive bacteria. However, a common system to these groups was discovered, the autoinducer 2. QS has implications in biofilm formation, where the application of metagenomic techniques to study these phenomena may be useful to understand the communication networks established by the different components of the community, and to discover new targets for microbial control. Here we present an *in silico* screening of QS proteins in all publicly available biofilm metagenomes from the JGI database. We performed sequence, conserved motifs, phylogenetic, and three-dimensional structure analyses of the candidates, resulting in an effective strategy to search QS proteins in metagenomes sequences. The number of QS proteins present in each sample, and its phylogenetic affiliation, was clearly related to the bacterial diversity and the origin of the biofilm. The samples isolated from natural habitats presented clear differences with those from artificial habitats. Interesting findings have been made in the abundance of LuxR-like proteins finding an unbalanced ratio between the synthases and the receptor proteins in Bacteroidetes bacteria, pointing out the existence of “cheaters” in this group. Moreover, we have shown the presence of the LuxI/R QS system in bacteria from the Nitrospira taxonomic group. Finally, some undescribed proteins from the HdtS family have been found in Gamma-proteobacteria.

## Introduction

*Quorum sensing* (QS) is a sophisticated cell to cell signaling mechanism mediated by small diffusible molecules called “autoinducers” that allow the populations to coordinate their behavior (Miller and Bassler, 2001; Bandara et al., 2012). This phenomenon is well studied in bacteria, where different QS systems have been described. In many Gram-negative bacteria there is a two components system integrated by a synthase (LuxI), responsible for the production of the QS molecules N-acyl homoserine lactones (AHLs), and a sensor receptor (LuxR), responsible for the detection of the molecules in the environment (Fuqua et al., 1994). In other kind of communication mediated by AHLs autoinducer-1 (AI-1) the receptor is a membrane-bond histidine kinase from the LuxN family (i.e., LuxN or AinR) and the synthesis of the QS molecule is not dependent on a LuxI-like enzyme, but on an enzyme from the LuxM family (i.e., LuxM or AinS) (Gilson et al., 1995; Milton, 2006). Finally, a third type of AHLs synthase was found in Gram-negative bacteria, the HdtS family that is neither a member of the LuxI nor of the LuxM families of proteins (Laue et al., 2000). In general, the substrates for AHL synthases are S-adenosyl-L-methionine (SAM) and an acyl-thioester in the form of an acyl-acyl carrier protein (ACP) intermediate of fatty acid biosynthesis, or for some acyl-HSL synthases acyl-Coenzyme A (acyl-CoA) (Christensen et al., 2014). In the case of HdtS, it is involved in both the acylation of SAM in AHL biosynthesis, and the acylation of lysophosphatidic acid in cell membrane biosynthesis, suggesting a dual function (Shih et al., 1999). Other QS molecules found as in other Gram-negative bacteria are the alkyl-hydroxy-quinolones. This is the case of *Pseudomonas aeruginosa* that synthesizes the signals 2-heptyl-3-hydroxy-4(1H)-quinolone (PQS) and 2-heptyl-4-quinolone (HHQ) via the cluster pqsABCDEH (Diggle et al., 2007). Moreover, this bacterium possesses two Lux-type QS systems that regulates numerous virulence genes. Later on, QS mechanisms mediated by the autoinducer-2 (AI-2; furanosyl borate diester) were discovered in Gram-positive and Gram-negative bacteria. In this mechanism the synthesis of the QS molecule is mediated by the product of the *luxS*-like genes that encode the (2S,4S)-2-methyl-2,3, 3,4-tetrahydroxy tetrahydrofuran-borate synthase. In addition, LuxS has a dual role in the cell acting as part of the activated methyl cycle pathway of some bacteria in which S-adenosylhomocysteine is recycled to recover S-adenosylmethionine (Pereira et al., 2013). The signal transduction is carried out by the family of soluble periplasmic AI-2 binding proteins LuxP (Hardie and Heurlier, 2008). There are many examples of bacteria that use at the same time the AI-1 and the AI-2 QS systems (Mok et al., 2003).

Gram-positive bacteria typically use small peptides as QS signal molecules (Dunny and Leonard, 1997). This system consists in a set of genes encoded in a cassette (i.e., ComACDE has been described in *Streptococcus pneumoniae*), which produces a propeptide that is exported and processed by extracellular proteases to the mature signaling peptide (Dunny and Leonard, 1997). This system also harbors a transporter for the exportation, and eventually, of the subsequent internalization of the peptide, which later is detected by an intracellular receptor of the Rap, NprR, PlcR, or PrgX protein families that binds directly to their specific signaling peptide and initiates a phosphorylation cascade regulating the transcription of certain genes (Dunny and Leonard, 1997). Later on, γ-butyrolactone was found as the QS molecule used by species of the genus *Streptomyces* (Polkade et al., 2016). In addition, some authors have described the communication between bacteria and eukaryotes (Barriuso et al., 2008), and have discovered the QS mechanisms in fungi (de Salas et al., 2015; Barriuso, 2016). On the other hand, other organisms have evolved strategies to inactivate the QS molecules from other microorganisms in a phenomenon called quorum quenching (Zhu and Kaufmann, 2013).

QS mechanisms have emerged among the most important aspects in the study of bacterial behavior, and are well characterized at the genetic and molecular levels due, among others, to their implication in biofilm formation and their possible application in antibacterial therapy to palliate the increasing antibiotic resistances (Rasamiravaka and El Jaziri, 2016). Biofilms are prevalent structures in many environments, and are formed by matrix-enclosed microbes that adhere to biological or non-biological surfaces. These adherent cells are frequently embedded within a self-produced extracellular polymeric matrix. This structured microbial community is a protected growth modality that allows bacteria to survive in hostile environments (Hall-Stoodley et al., 2004). The maturation of these aggregated microbial cells is mediated by changes in cell phenotype and physiology regulated by QS, among a variety of mechanisms (Jabra-Rizk et al., 2006). For these reasons, biofilms are an ideal habitat for ecological studies (Davey and O’toole, 2000).

On the other hand, metagenomics has experienced a great development during the last decade thanks to massive DNA sequencing, contributing to revise our view on microbial biodiversity and microbial communities (Barriuso et al., 2010, 2013; Barriuso and Martínez, 2015). There are several public databases with a huge amount of this information available, among which are MG-RAST and IMG/M (Markowitz et al., 2012). Moreover, the advances in bioinformatics methodologies are allowing the discovery of new genes and proteins in these datasets (Barriuso et al., 2011).

In the present work we carried out a sequential bioinformatic screening of publicly available metagenomes from biofilm samples of different environments to look for bacterial QS proteins. The candidates were selected taking into account the annotation in the databases, sequence similarity, conserved motifs, phylogenetic affiliation, and three-dimensional structure models of the putative proteins. Moreover, the number of QS genes and their phylogenetic affiliation were discussed on the basis of the habitat, their ecological interactions and the evolutionary scenario of the QS systems. These studies would be useful to increase the knowledge into biofilms functioning and for the development of potential antimicrobial therapies.

## Materials and Methods

Metagenomes were analyzed from the server of the Joint Genome Institute, IMG/M^1^. This public genomic data repository contains more than 8240 metagenomes from different environments; the metagenomes were filtered for those containing the term “biofilm” in their description (**Supplementary Table S1**). Among the 22 biofilm metagenomes found, 11 corresponded to samples isolated from natural groundwater areas, while 11 corresponded to habitats modified by the human action (**Supplementary Table S1**). The total number of protein coding sequences analyzed was 9.801.774.

We explored QS proteins from all known bacterial QS systems in the “biofilm metagenomes” using two strategies (**Supplementary Table S2**). In first place, we used the “search by term” option filtering by “Gene Product Name” at the IMG/M server using 51 different queries: “quorum, sensing, quorum sensing, Autoinducer, Homoserine lactone, AHL, signal peptides, cyclic peptide, AI-1, AinS, HdtS, AI2, Furanosyl borate diester, Quinolone, PQS, HHQ, AHQ, butyrolactone, LuxI, LasI, YenI, EsaI, RhlI, HdtS, LuxR, LasR, TraR, RhlR, LuxM, LuxLM, AinS, AinR, LuxS, LuxP, pqsA, pqsB, pqsC, pqsD, pqsE, pqsH, Gamma-butyrolactone, ComA, ComC, ComD, ComE, ComR, ComX, PlcR, Papr7, NprR, Rap.” In the second strategy, we analyzed the sequence similarity of putative proteins in the assembled metagenomes using blastp (cut-off value 1e^-10^) taking as reference QS proteins well characterized from different families. Accession numbers of these proteins are showed in **Supplementary Table S2**. When reference sequences from different bacterial phyla were available the blastp analysis was performed by using all sequences available to maximize the scrutiny. This is the case of LuxP, analyzed by using reference sequences from alpha and Gamma-proteobacteria species, and the case of pqsH, analyzed using sequences from Proteobacteria, Actinomycetes and Firmicutes species.

The selected candidate sequences from the each protein family were grouped into a FASTA file and filtered; sequences with less than 50 amino acids were deleted and the rest of the sequences were subjected to a search for conserved motifs using InterProScan v5 sequence search with default parameters^2^ (Jones et al., 2014). The sequences which did not matched to the selected family were eliminated, and the rest of the candidates were subjected to multiple sequence alignment, including representative sequences of each family, using MUSCLE algorithm with the following parameters: Gap open -2.9, Gap extend 0, Hydrophobicity multiplier 1.2, UPGMB, min diag length 24 (Edgar, 2004). Subsequently, a phylogenetic tree was built using the following parameters: Maximum-Likelihood method, JTT model, uniform rates, all sites, NNI, BioNJ) and evaluated with a bootstrap value of 1000 (MEGA7.0.21). Sequences grouping with the model proteins in the trees were selected for further analyses. The taxonomical affiliation of each candidate was deduced from the BLAST and the phylogenetic analyses.

In the case of HdtS family, a three-dimensional model of the most interesting candidates was generated with the programs implemented by the automated protein homology-modeling server SWISS-MODEL (Swiss Institute of Bioinformatics^3^; Biasini et al., 2014). The template used was the crystal structure of the related enzyme 1-acyl-sn-glycerophosphate lysophosphatidic-acid (LPA) acyltransferase, PlsC, from *Thermotoga marítima* (5KYM). The quality of the four models was good with a QMEAN scores between -2.01 and -3.94. They were exhaustively analyzed using PyMol 1.1^4^ (Kozlikova et al., 2014).

Finally, the number of candidates was normalized as a function of the genome size assembled (nt) (Romero et al., 2012) to avoid bias due to the genome size. The normalized value is the results of dividing the number of QS proteins by the size (nt) of each assembled metagenome. Excel matrixes and graphical representations were built with the candidate’s distribution in each metagenome and the taxonomical affiliation of each protein. The matrix with the normalized number of candidates was used for multivariate analysis at the server ClustVist^5^, which was represented in a Heatmap correlating the results with the ecological niche of each metagenome. The clustering distance was calculated using the Manhattan method, and the linkage method selected was the Average (Metsalu and Vilo, 2015).

## Results and Discussion

Biofilms are structures very resistant to environmental fluctuations and to antimicrobial therapies (Hall-Stoodley et al., 2004). The formation of these structures implies the coordination of different populations of bacteria to acquire the appropriate spatial disposition and to organize their functioning. In this sense, QS mechanisms allow the communication of different species of microorganisms (Jabra-Rizk et al., 2006). Because of the high abundance of bacteria and their biotechnological and clinical implications biofilms are an ideal environment for the study of QS.

In this work, we employed two different strategies to look for QS proteins in 22 data sets from 18 metagenomes isolated from biotechnologically or environmentally relevant biofilms (**Supplementary Table S1**). Putative QS proteins were found in all the metagenomes analyzed except for samples Ga0052129, JGI12104J13512, RS9, and Ga0052186; in the first three cases probably because of the low number of reads and in the later because of the low diversity of bacteria (**Supplementary Table S1**). This is the opposite case to metagenome LI09_3 that has higher number of predicted coding-sequence than expected by its size in base pairs (**Supplementary Table S1**).

To discard a possible imbalance in the number of candidates obtained in each sample caused by the size of each metagenome we performed a search of two components of the complex SecYEG, the proteins SecE and SecG. These proteins are between 75 and 125 amino acids in length and form a protein-conducting channel, in a ratio 1:1, in the Sec secretion machinery present in most bacteria (Green and Mecsas, 2016). The “search by term” of SecE and SecG yielded approximately the same number of protein candidates in each metagenome (**Supplementary Table S3**).

The two search strategies used rendered different number of hits (**Supplementary Table S2**). A total of 2470 candidate proteins were obtained using the “search by term” strategy, from which 548 hits remained after filtering. These “false positives” were produced because of the use of very general terms, such as “Quorum” and “sensing,” and because of the incorrect annotations of some proteins at the IMG pipeline. This is the case of PEP-CTERM/exortases, sugar acyltransferases (GNAT-family), transposase, hybrid histidine kinase, and transcriptional regulators that were annotated as Lux proteins. The search with the term “homoserine lactone” rendered 31 hits after filtering that corresponded to LuxI proteins, while the search with the terms “AI2” or “LuxS” rendered the same 145 proteins from the LuxS family. Furthermore, the term “Autoinducer” generated a high number of candidates, but all of them corresponded to LuxS or LuxR protein families (**Supplementary Table S2**). The second strategy consisted in a blastp analysis using as query 37 reference protein sequences from species with different phylogenetic affiliations. This search rendered 996 candidates (**Supplementary Table S2**) using a cut-off value of 1e^-10^ that typically produces matches with a sequence identity higher than 20%, avoiding the presence of “false positives.” However, the drawback of this strategy is the low availability of reference sequences among the different protein families.

**FIGURE 1:**
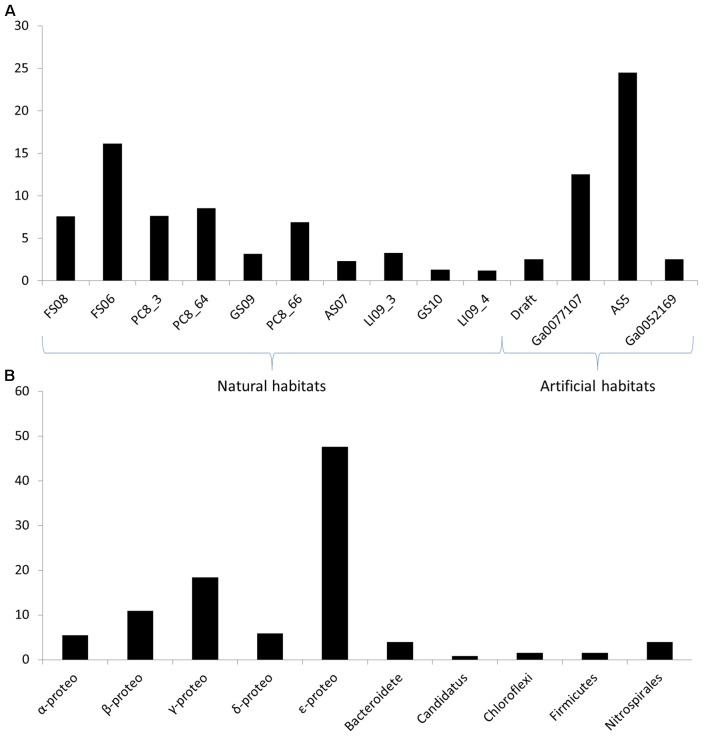
**(A)** Normalized percentage of *quorum sensing* (QS) protein candidates in each metagenome referred to the total number of hits. The normalized value is the results of dividing the number of QS protein candidates by the size (nt) of each assembled metagenome. **(B)** Number of hits of the QS protein candidates in each taxonomic group.

We mixed the results of both strategies to maximize the study. When analyzing the number of hits per metagenome, it does not seem to be related to the sequencing depth of each sample. The metagenomes with higher number of reads were LI09_4, GS10, GS09, and PC8_66, respectively (**Supplementary Table S1**), while the metagenomes with higher number of QS candidate sequences were: FS06, PC8_66, PC8_64, and PC8_3, respectively (**Figure 1** and **Supplementary Table S4**). This may be due to the abundance of species in each biofilm, related directly with the ecological niche. Metagenomes FS (Fissure Spring), GS (Grotta Sulfurea), LI (Lago Infinito), PC (Pozzo dei Cristalli), and RS (Ramo Sulfureo) come from the Frasassi cave systems in Italy (Macalady et al., 2006), which is a lithoautotrophic ecosystems characterized by sulfur-oxidizing biofilms (Macalady et al., 2007) with high abundance of filamentous Epsilon-proteobacteria that are among the principal biofilm architects in Frasassi streams (Hamilton et al., 2014). The QS proteins found in these metagenomes were mainly from Epsilon-proteobacteria with the AI-2 system (**Supplementary Table S4**). However, the metagenomes from the Acquasanta Terme systems (AS05 and AS07), also in Italy, had a low number of hits despite they have a similar bacterial community structure (Jones et al., 2010).

**FIGURE 2:**
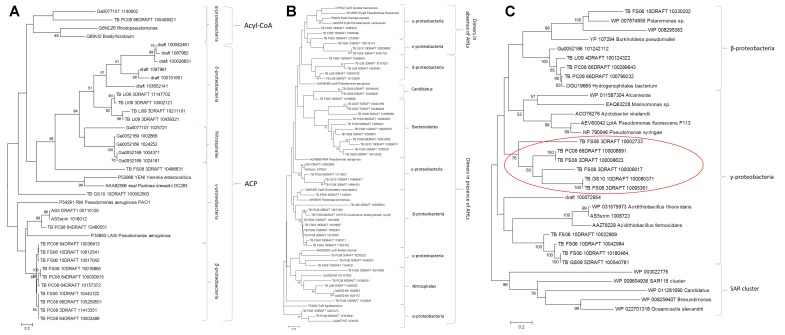
Phylogenetic analysis of the candidates from the LuxI family **(A)**, LuxR family **(B)**, and the HtdS family **(C)**. Only bootstrap values above 50% are shown.

The selected candidates from each family, together with the reference sequences, were subjected to phylogenetic analysis. The trees of the protein families involved in QS system that uses AHLs as signaling molecules (LuxI, HdtS, and LuxR) are shown in **Figure 2**. In general, there is a great abundance of Proteobacteria as expected, since the AI-1 systems are exclusive from Gram-negative bacteria. The Lux-type systems have been experimentally characterized in more than 70 different species in the phylum Proteobacteria (Nasuno et al., 2012).

In the phylogenetic reconstructions of the LuxI proteins (**Figure 2A**), at the top of the tree, it can be observed a branch formed by two reference sequences from Alpha-proteobacteria and two candidate proteins. Those sequences correspond to AHL synthases that use preferentially acyl-CoA as substrate (Christensen et al., 2014), while the other sequences in the tree grouped with reference sequences that use preferentially ACP as substrate. In the phylogeny of the LuxR proteins (**Figure 2B**), there is a clear division between the group of proteins that are active and form dimers in the presence of AHLs and the group of proteins that are active and form dimers in the absence of AHLs (Lerat and Moran, 2004). There is also a third group formed by Delta-proteobacteria, bacteria Candidatus, and Bacteroidetes that did not group with any reference sequence. Interestingly, in the trees from LuxI and LuxR there are sequences from the phylum Nitrospirae, and in the case of the tree from LuxR also members of the phylum Bacteroidetes. This fact suggests that not only Proteobacteria (Nasuno et al., 2012), but also a diverse array of as-yet-uncultivated bacteria likely possess LuxI/R-type QS systems as shown by other authors (Biswa and Doble, 2013). In addition, the presence of Bacteroidetes with LuxR genes that do not have the corresponding synthase from the families described so far (LuxI, LuxM, or HdtS) it is very interesting (**Supplementary Table S4**). It cannot be ruled out the presence of a yet unknown AHLs synthase from a new family that share no homology with the known sequences in this group of bacteria. However, there has been described in other bacteria that part of the population is able to synthetize and sense the AHLs, while other part, so called “cheaters,” is only able to sense the AHLs taking advantage of the investment of the others. The presence of these “LuxR solos” is of great importance in the population dynamics (Subramoni et al., 2015). In the case of *Salmonella typhimurium* it was shown that SdiA can detect AHL produced by other species, suggesting that this protein could be involved in recognizing the presence of foreign bacteria, despite its role is still controversial (LaSarre and Federle, 2013).

HdtS-like proteins have been proposed to constitute a third family of AHL synthases, and an HdtS homolog from *Pseudomonas fluorescens* F113 has been shown to catalyze production of C6-, C10-, and 3-OH-C14:1-HSL (Laue et al., 2000). The phylogenetic study of the HdtS family (**Figure 2C**) showed three clusters, one with sequences belonging to the Beta-proteobacteria group, a second one with sequences derived from other metagenomics studies (SAR11, SAR86, and SAR116) (Doberva et al., 2015), and another formed by Gamma-proteobacteria. The later was divided in three sub-groups, one including seven reference sequences from *Pseudomonas*, *Azotobacter*, *Marinomonas*, and *Alcanivorax*, other including six candidates that grouped with *Acidithiobacillus* sequences, and other group including six candidates that did not group with any known reference sequence (indicated with a red circle in **Figure 2C**). Two candidates from this new group of enzymes were selected for further analyses (FS08_3DRAFT_10095361 and PC08_66DRAFT_100008891). This selected sequences presented an identity of 34 and 42%, respectively with the model protein from *P. fluorescens* F113 HdtS LptA (AEV600421). HdtS family is homologous to neither LuxI- or AinS-type proteins but rather to the LPA acyltransferase family, which are generally involved in the biosynthesis of the phospholipid precursor phosphatidic acid, a critical intermediate in cell membrane biosynthesis (Laue et al., 2000). HdtS proteins possesses two motifs, NHQS and PEGTR, which are highly conserved in prokaryotic and in eukaryotic LPA acyltransferases (Robertson et al., 2017). The three-dimensional models of the two candidates and the *P. fluorescens* F113 reference sequence (**Supplementary Figure S1**) showed the same folding, with the presence of a two-helix motif in the N-terminal, linked to an acyltransferase domain that contains the catalytic core of the enzyme. The models presented the same disposition of the active residues His84, Asp89, Lys105, and Arg159 as the template structure (PlsC from *T. marítima*) (Robertson et al., 2017), but with the Asp89 substituted by a glutamic acid (Glu). The topology of the surface, the substrate (LPA)-binding groove and the entrance to the acyl-chain-selectivity tunnel, were similar in the three proteins (**Supplementary Figure S1**).

On the other hand, the phylogenetic tree from the 145 candidate sequences from LuxS proteins (data not shown) showed how these proteins were very close phylogenetically. Most of the candidates belong to the Epsilon-proteobacteria phylum, while there were few sequences from the Firmicutes and the Chloroflexi phyla (**Supplementary Table S4**). In the case of LuxP candidates there were few sequences (7) most of them grouping with Alpha-proteobacteria and two of them grouping with Gamma-proteobacteria reference sequences (**Supplementary Table S4**). Furthermore, no candidates from the quinolones or the Gram-positive bacteria QS systems were found in the metagenomes screening. This is probably due to lower abundance of these bacteria compared to Gram-negative in these habitats, the absence of annotation for this family in the data base, and the low number of reference sequences available to carry out the BLAST analysis.

**FIGURE 3:**
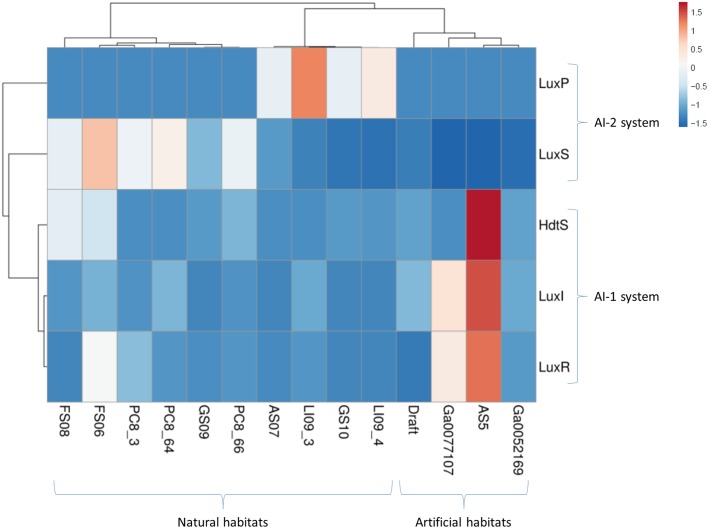
Heatmap representation of the number of QS protein candidates found in each metagenome.

The distribution of the QS proteins was studied in each genome using the normalized number of candidates. The **Figure 1** and **Supplementary Table S5** shows the abundance of the candidates from the families LuxI, LuxR, HdtS, LuxS, and LuxP. Multivariate analysis of the matrix of the candidate’s abundance was performed to correlate the number of QS genes to the habitat. **Figure 3** shows a statistical correlation between the different families of proteins and the habitat of each metagenome. The samples from groundwater habitats grouped together in the left part of the heatmap, while artificial ecosystems grouped together at the right side of the heatmap according to the QS systems present in each metagenome (**Figure 3**). LuxS proteins that may have other function rather than the synthesis of QS molecules may cause some noise in the analysis of abundance (Mok et al., 2003), thus the analysis was repeated with the abundance of the candidates from the AI-1 system and the AI-2 system independently showing similar results and running out the interference in the analysis of the LuxS candidates.

The AI-2 system is more represented in the groundwater habitats, rich in Epsilon-proteobacteria. Furthermore, the components of the AI-1 and the AI-2 QS systems grouped together in the other axis of the heatmap. The ecological interactions among the members of the each community seems to be complex and mediated by different QS molecules that may be recognized by different species, while other bacteria carrying “LuxR solos” are able to sense and respond to the environment signals, but not to produce it.

## Conclusion

In this work we have developed an effective tool to look for QS genes in genomes and metagenomes, analyzing the advantages and disadvantages of each strategy. We have analyzed in depth the candidates obtained revealing their phylogenetic affiliation, their properties within each family, and in the case of the HdtS family, we have described a novel group of candidates. Finally, we have studied the distribution of the QS proteins in each metagenome, showing a correlation between the QS systems and the habitat. These results pave the way to perform wet-lab experiments to increase the understanding of this communication processes in bacterial biofilms, which will be useful in the development of new antimicrobial therapies.

## Author Contributions

JB designed the work, performed the bioinformatics screening, and prepared the manuscript. MM has collaborated in the interpretation of the results and preparation of the manuscript.

## Conflict of Interest Statement

The authors declare that the research was conducted in the absence of any commercial or financial relationships that could be construed as a potential conflict of interest.

## Footnotes

1 http://img.jgi.doe.gov

2 https://www.ebi.ac.uk/interpro/

3 https://swissmodel.expasy.org/

4 http://pymol.org/

5 http://biit.cs.ut.ee/clustvis/

## Supplementary Material

FIGURE S1 Three-dimensional models of the protein candidates from the HtdS family. **(a)** *Pseudomonas fluorescens* F113, **(b)** FS08_3DRAFT_10095361, and **(c)** PC08_66DRAFT_100008891. The upper panel shows the overall structure of the proteins with the N-terminal two-helix motif colored orange. Functionally important residues are highlighted in red. The lower panel shows a representation of the surface of the proteins with the substrate lysophosphatidic-acid (LPA)-binding groove and the entrance to the acyl-chain tunnel marked with an arrow.

TABLE S1 Properties of the metagenomes analyzed from different biofilms. Eleven corresponded to samples taken from natural niches and 11 from habitats modified by human action.

TABLE S2 Number of candidates obtained in the search of *quorum sensing* (QS) proteins in 22 metagenomes from biofilm samples.

TABLE S3 Number of SecE and SecG protein candidates obtained using the “search by term” in the different metagenomes from biofilm samples deposited at the JGI database.

TABLE S4 Abundance of QS protein candidates in each metagenome and in each phylogenetic group.

TABLE S5 Normalized number of QS protein candidates in each metagenome.
