# Supplementary figures and images for: In Silico Analysis of the Quorum Sensing Metagenome in Environmental Biofilm Samples
Source: Front Microbiol. 2018 Jun 7;9:1243. doi: 10.3389/fmicb.2018.01243 (PMC6000730; doi:10.3389/fmicb.2018.01243)

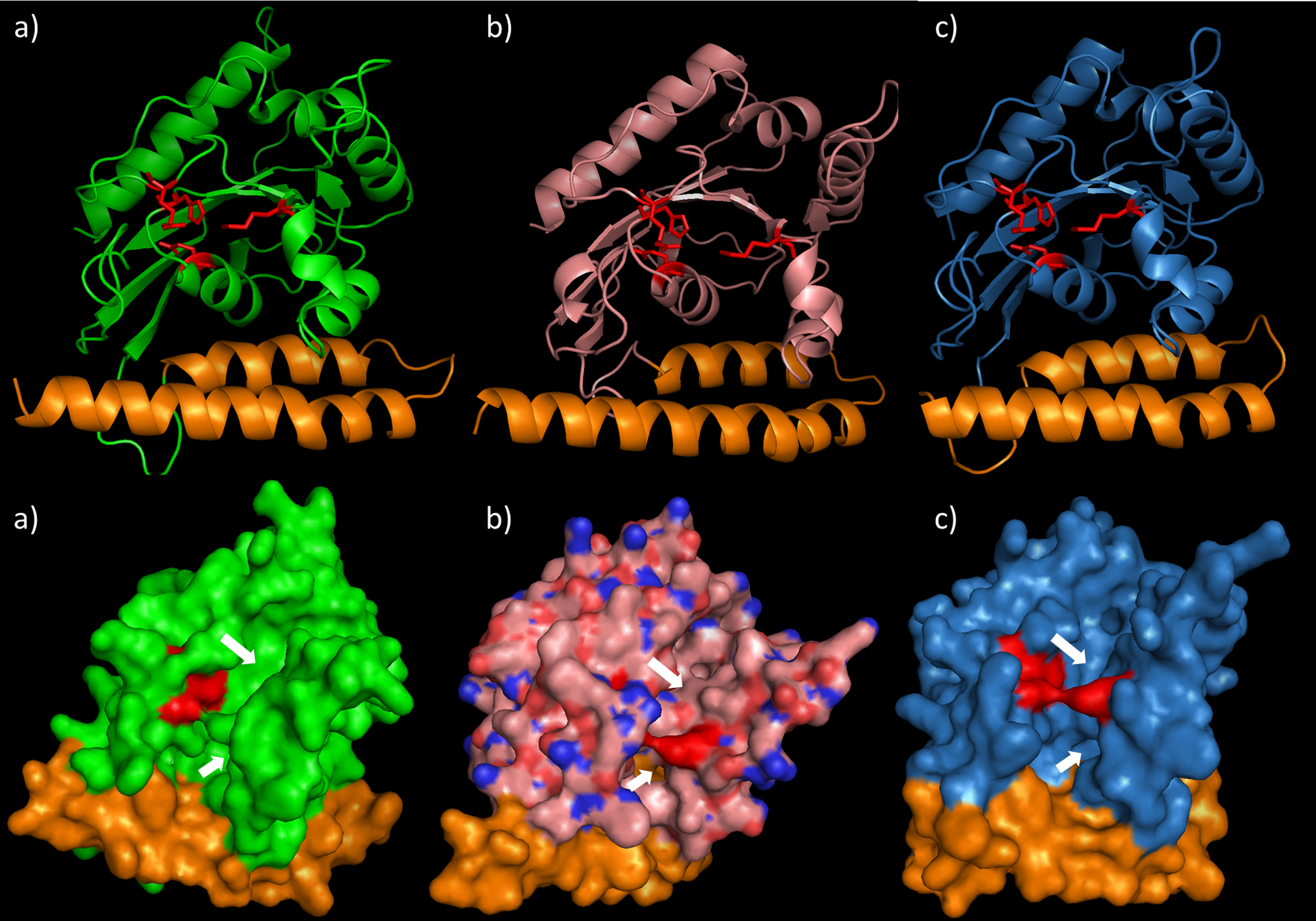

Supplement: FIGURE S1 — Three-dimensional models of the protein candidates from the HtdS family. (a) Pseudomonas fluorescens F113, (b) FS08_3DRAFT_10095361, and (c) PC08_66DRAFT_100008891. The upper panel shows the overall structure of the proteins with the N-terminal two-helix motif colored orange. Functionally important residues are highlighted in red. The lower panel shows a representation of the surface of the proteins with the substrate lysophosphatidic-acid (LPA)-binding groove and the entrance to the acyl-chain tunnel marked with an arrow. [file Image_1.TIF]
