# Supplementary material for: In Silico Analysis of the Quorum Sensing Metagenome in Environmental Biofilm Samples
Source: Front Microbiol. 2018 Jun 7;9:1243. doi: 10.3389/fmicb.2018.01243 (PMC6000730; doi:10.3389/fmicb.2018.01243)
Supplement: TABLE S1 — Properties of the metagenomes analyzed from different biofilms. Eleven corresponded to samples taken from natural niches and 11 from habitats modified by human action. [file Table_1.DOCX]

**Table S1.** Properties of the metagenomes analyzed from different biofilms. Eleven corresponded to samples taken from natural niches and eleven from habitats modified by human action.

| **Nº** | **Study name** | **Code** | **IMG Genome ID** | **Habitat** | **Genome Size assembled (bp)** | **Coding-sequences** | **Isolation localization** |
| --- | --- | --- | --- | --- | --- | --- | --- |
| 1 | Pink biofilm microbial community from flowing acid mine drainage | Ga0052169 | 3300003104 | Acid Mine Drainage | 10756372 | 12840 | Richmond mine, Iron Mountain, California, USA |
| 2 | Acid Mine Drainage (AMD) microbial and viral communities | Ga0052129 | 3300003083 | Acid Mine Drainage | 2632775 | 2707 | Richmond mine, Iron Mountain, California, USA |
| 3 | Bioreactor community from anode biofilm microbial fuel cells | Ga0052186 | 3300003154 | Bioreactor | 290214330 | 447284 | North City Water Reclamation Plant (San Diego, CA, USA) |
| 4 | Crenothrix polyspora biofilm communities | Ga0077107 | 3300005627 | Drinking water | 24508778 | 47380 | Wolfenbuettel waterworks, Germany |
| 5 | Fissure Spring (below trough), Frasassi Caves, Italy | GS09 | 3300000234 | Groundwater | 539638524 | 1018313 | Targeted biofilms from Grotta Sulfurea, Alcove, Frasassi Caves, Italy |
| 6 | Grotta Sulfurea, Talus Pool, Frasassi Caves, Italy | GS10 | 3300000230 | Groundwater | 581077747 | 1202373 | Targeted biofilms from Grotta Sulfurea, Talus Pool, Frasassi Caves, Italy |
| 7 | Acquasanta Terme, Italy | FS06 | 3300000233 | Groundwater | 242610534 | 467245 | Targeted biofilms from Fissure Spring, Frasassi Caves, Italy |
| 8 | Fissure Spring, Frasassi Caves, Italy | FS08 | 3300003414 | Groundwater | 1498780 | 1199 | Targeted biofilms from Fissure Spring (below trough), Frasassi Caves, Italy |
| 9 | Fissure Spring, Frasassi Caves, Italy | FS08 | 3300000236 | Groundwater | 181303868 | 396513 | Targeted biofilms from Fissure Spring (below trough), Frasassi Caves, Italy |
| 10 | Grotta Sulfurea, Talus Pool, Frasassi Caves, Italy | LI09_3 | 3300000229 | Groundwater | 281841843 | 717718 | Targeted biofilms from Lago Infinito, wall in 7.1m depth, Frasassi Caves, Italy |
| 11 | Lago Infinito, wall in 7.1 m depth, Frasassi Caves, Italy | LI09_4 | 3300000231 | Groundwater | 654101350 | 1502367 | Targeted biofilms from Lago Infinito, wall in 6.8 m depth, Frasassi Caves, Italy |
| 12 | Lago Infinito, wall in 6.8 m depth, Frasassi Caves, Italy | PC8_3 | 3300000235 | Groundwater | 252007248 | 509216 | Pozzo dei Cristalli, Frasassi Caves, Italy |
| 13 | Pozzo dei Cristalli, Frasassi Caves, Italy | PC8_64 | 3300000232 | Groundwater | 270071044 | 563728 | Pozzo dei Cristalli, Frasassi Caves, Italy |
| 14 | Pozzo dei Cristalli, Frasassi Caves, Italy | PC8_66 | 3300000228 | Groundwater | 448373019 | 966683 | Pozzo dei Cristalli, Frasassi Caves, Italy |
| 15 | Hydrocarbon resource environments microbial communities | Draft | 3300002697 | Industrial wastewater | 18136085 | 366985 | Fort McMurray, Alberta, Canda |
| 16 | Hydrocarbon resource environments microbial communities | Draft | 3300002702 | Industrial wastewater | 289715476 | 619401 | Fort McMurray, Alberta, Canda |
| 17 | Subaerial biofilm microbial communities from sulfidic caves | AS5 | 2199352028 | Rock-dwelling (subaerial) | 7103528 | 13668 | Grotta Nuova di Rio Garrafo, near Acquasanta Terme, Italy |
| 18 | Subaerial biofilm microbial communities from sulfidic caves | AS5 | 3300000825 | Rock-dwelling (subaerial) | 8590020 | 17399 | Grotta Nuova di Rio Garrafo, near Acquasanta Terme, Italy |
| 19 | Wolfenbuettel Waterworks, Germany | AS07 | 3300000227 | Groundwater | 300437395 | 532506 | Acquasanta Terme, Italy |
| 20 | Subaerial biofilm microbial communities from sulfidic caves | RS9 | 2199352029 | Rock-dwelling (subaerial) | 6725589 | 12672 | Frasassi cave system, Italy |
| 21 | Subaerial biofilm microbial communities from sulfidic caves | RS9 | 3300000824 | Rock-dwelling (subaerial) | 6740218 | 10806 | Frasassi cave system, Italy |
| 22 | Wastewater bioreactor microbial communities from Singapore and Univ of Illinois at Urbana, that are terephthalate-degrading | JGI12104J13512 | 3300001095 | Wastewater bioreactor | 217084359 | 372771 | National University of Singapore, Singapore |
