# Supplementary material for: In Silico Analysis of the Quorum Sensing Metagenome in Environmental Biofilm Samples
Source: Front Microbiol. 2018 Jun 7;9:1243. doi: 10.3389/fmicb.2018.01243 (PMC6000730; doi:10.3389/fmicb.2018.01243)
Supplement: TABLE S2 — Number of candidates obtained in the search of quorum sensing (QS) proteins in 22 metagenomes from biofilm samples. [file Table_2.DOCX]

**Table S2.** Number of candidates obtained in the search of QS proteins in 22 metagenomes from biofilm samples.

| **Protein/term** | **Function/accession number** | **Search by term number of hits** | **Search by term number of hits after filtering** | blastp **number of hits** | blastp **number of hits after filtering** |
| --- | --- | --- | --- | --- | --- |
| Quorum | - | 503 | - | - | - |
| sensing | - | 804 | - | - | - |
| Quorum Sensing | - | 409 | - | - | - |
| Autoinducer | - | 259 | 186 |  |  |
| Homoserine lactone | - | 73 | 31 | - | - |
| AHL | - | 0 | - | - | - |
| signal peptides | - | 0 | - | - | - |
| cyclic peptide | - | 0 | - | - | - |
| AI-1 | - | 0 | - | - | - |
| AinS | - | 0 | - | - | - |
| HdtS | - | 0 | - | - | - |
| AI2 | - | 163 | 145 | - | - |
| Furanosyl borate diester | - | 0 | - | - | - |
| Quinolone | - | 0 | - | - | - |
| PQS | - | 0 | - | - | - |
| HHQ | - | 0 | - | - | - |
| AHQ | - | 0 | - | - | - |
| Butyrolactone | - | 0 | - | - | - |
| LuxI | AHL synthase (P35328) | 0 | - | 11 | 11 |
| LasI | AHL synthase (BAT66620) | 0 | - | 13 | 13 |
| YenI | AHL synthase (P52988) | 0 | - | 1 | 1 |
| EsaI | AHL synthase (AAA82096) | 0 | - | 1 | 1 |
| RhlI | AHL synthase (P54291) | 0 | - | 12 | 12 |
| HdtS | AHL synthase (AEV60042) | 0 | - | 17 | 17 |
| LuxR | AHL receptor (AAQ90229) | 96 | 41 | 3 | 3 |
| LasR | AHL receptor (BAA06489) | 0 | - | 29 | 29 |
| TraR | AHL receptor (P54294) | 0 | - | 3 | 3 |
| RhlR | AHL receptor (ACI42868) | 0 | - | 34 | 34 |
| LuxLM | AHL synthase (AAG24822) | 0 | - | 0 | - |
| AinS | AHL synthase (YP_204420) | 0 | - | 0 | - |
| AinR | AHL receptor (YP_204419) | 0 | - | 256 | 0 |
| LuxS | S-ribosylhomocysteine lyase (KPU55950, AAG28749, OFC89811, ANW92499, CAL35313, WP_028584559, CDB53682 and AAD17292) | 163 | 145 | 145 | 145 |
| LuxP | AI-2 binding protein  (CUK26632, YP_204090 and CCH48811) | 0 | - | 8 | 7 |
| pqsABCD | PQS biosynthesis protein (BAT67082) | 0 | - | 0 | - |
| pqsH | PQS biosynthesis protein (BAT65374) | 0 | - | 54 | 0 |
| Arpa-Like receptor | Gamma-Butyrolactone Receptor (pdb 1UI6) | 0 | - | 13 | 0 |
| ComACDER and X | Streptococcus signal peptide biosynthesis and transport (AAA69510, ACT32372, AAG18644, AAC44895, 5FD4 and AAF82173) | 0 | - | 10 | 0 |
| Papr7 / PlcR and NprR | Bacillus signal peptide and receptors (pdb 3U3W, 2QFC, 4GYO and 4GPK) | 0 | - | 26 | 0 |
|  | Bacillus signal peptide and receptor (pdb) |  |  |  |  |
| **Total** |  | **2470** | **548** | **1320** | **996** |

Two strategies were used for the in silico mining of the public metagenomes. The first strategy consisted in the extraction of sequences “searching by term” into the annotation of the database. The second strategy consisted in comparison of reference sequences with the proteins present in the database by means of blastp (cut-off value 1e^-10^).
